# DISC1 and Huntington's Disease – Overlapping Pathways of Vulnerability to Neurological Disorder?

**DOI:** 10.1371/journal.pone.0016263

**Published:** 2011-01-26

**Authors:** Ruth Boxall, David J. Porteous, Pippa A. Thomson

**Affiliations:** Medical Genetics Section, Molecular Medicine Centre, Institute of Genetics and Molecular Medicine, University of Edinburgh, Western General Hospital, Edinburgh, United Kingdom; Hospital Vall d'Hebron, Spain

## Abstract

We re-annotated the interacting partners of the neuronal scaffold protein DISC1 using a knowledge-based approach that incorporated recent protein interaction data and published literature to. This revealed two highly connected networks. These networks feature cellular function and maintenance, and cell signaling. Of potentially greatest interest was the novel finding of a high degree of connectivity between the DISC1 scaffold protein, linked to psychiatric illness, and huntingtin, the protein which is mutated in Huntington's disease. The potential link between DISC1, huntingtin and their interacting partners may open new areas of research into the effects of pathway dysregulation in severe neurological disorders.

## Introduction

Disrupted in Schizophrenia, *DISC1*, is one of the most convincing candidate genes for schizophrenia and related major mental illness [1]. DISC1 is a scaffold protein that interacts with multiple neurodevelomental, cytoskeletal and signaling proteins [2], several of which have been independently associated with major psychiatric illness [1]. Here, we update a previous analysis of the Disrupted in Schizophrenia, DISC1, interactome [2], seeking emergent evidence for novel linkages to other pathogenic pathways. The re-analysis of this dataset has revealed a strong network linking DISC1 and huntingtin protein, which is mutated in Huntington's disease [3], consistent with DISC1 and huntingtin regulating convergent pathways. We hypothesise that mutation in both DISC1 and HTT impact on a common pathway underpinning major psychiatric illness. It will be important now to test the effect of pathological mutation and genetic variation with the normal range in DISC1, HTT and intermediate genes on this convergent pathway.

In 2007, Carmargo et al. reported the results of a series of yeast two-hybrid screens using DISC1 and key interactors: CDC5L, CDK5RAP3, CEP63, DTNBP1, NDEL1, SEC3L1, SH3BP5, TNIK, TRAF3IP1 [2]. Their network analysis implicated DISC1 as a hub or scaffold protein with critical roles in cytoskeletal stability and organisation, intracellular transport, cell-cycle/division and synaptic function. Here, we revisit the question of DISC1 function through network analysis and identify multiple links to huntingtin (HTT), suggesting an overlapping pathway of vulnerability between DISC1-related psychiatric disorders and Huntington's disease, a neurodegenerative disorder characterized by a deterioration in motor, cognitive, and emotional function.

## Results

The protein set originally identified by Camargo et al (see Supplementary Information Table 3 within Camargo et al [2]) was subjected to Ingenuity Pathway Analysis (IPA, Ingenuity Systems), a knowledge-based functional annotation programme. Of the 150 genes in the raw gene list, 139 were mapped within the knowledge-base and 133 were network eligible. Network sizes were limited to a maximum of 140 molecules. Only direct interactions were permitted, however data sources used included Additional interactions, Ingenuity Expert Findings, MicroRNA-mRNA interactions, or Protein-protein interactions. Two extensive and highly connected, and thus high scoring, networks were identified: Cellular Function and Maintenance, Cell Cycle, Cellular Assembly and Organization, with an IPA score of 205 containing 96 key molecules (Figure S1); and Cell Signaling, DNA Replication, Recombination, and Repair, Nucleic Acid Metabolism, with an IPA score of 79 containing 49 key molecules (Figure S2). Of the molecules not in the original dataset that were added by the program to create these networks, several, such as PDE4D and FEZ1 are well documented DISC1 interactors [2], as well as DISC1 itself, were identified successfully.

The highest scoring network, Cellular Function and Maintenance, Cell Cycle, Cellular Assembly and Organization (Figure S1), contained hub molecules (defined as those with greater than 10 connections) which included cytoskeletal components: tubulin, actin, dynactin, the NDEL1/PAFAH1B1 (also known as LIS1) complex, as well as 14-3-3 proteins (including YWHAZ), the exocyst complex, CDC5L and TNIK. This network contained very few proteins not on the original input list, and did not include DISC1. Proteins added from the knowledge-base included: REM1, CLIC5, and the NMDA receptor pathway, suggesting that these molecules may also form part of the wider DISC1 interactome. This may reflect the recent report that DISC1 regulates the activation of Rac1 in response to NMDA receptor activation [4], which is also reflected in the addition of NMDA receptors and RAC1 to the second network, and indicates involvement of this complex as a direct link between the interactors AKAP9 and SMC3.

In the second highest scoring network, Cell Signaling, DNA Replication, Recombination, and Repair, Nucleic Acid Metabolism (Figure S2), the updated knowledge-base introduced several new molecules. These included PDE4D and FEZ1, which are linked to DISC1 through both protein interaction and functional experimental data [5]–[8]. Unsurprisingly, DISC1 formed a major hub protein within this network with 44 connecting proteins. What was striking, however, was the addition of the huntingtin protein (HTT) as an extremely well connected hub protein with 57 connections. No direct connection was made between DISC1 and HTT, but numerous proteins, both those on the input list and those added from the current knowledge-base, provided single step connections between these two neurologically important proteins. Statistical analysis of the overlap in interacting proteins identified in this IPA analysis gave a significant enrichment of DISC1 interactors within the proteins connected to HTT in Network 2 (P<0.001). To avoid any bias that may result from building this interaction network from DISC1 interactors, we have further analysed the lists of interactors of DISC1 and HTT from the EBI IntAct database (www.ebi.ac.uk/intact/) and again found significant enrichment of DISC1 interactors in the list of HTT interactors (P = 0.007). Known DISC1 interactors linking DISC1 to HTT in the second network included: FEZ1, IMMT, DPYSL2 (also known as CRMP2), GPRASP2, GNB1 and PDE4B. These are proteins with key functions in mitochondrial activity and trafficking, axon outgrowth and neuronal signaling. KIAA1377 was added to this network from the knowledge-base. KIAA1377, which interacts with FEZ1, IMMT, DISC1 and HTT, is localised in the midbody and believed to play a critical role in cytokinesis progression [9]. Other hub proteins in this network, and identified from the knowledge-base include: the NMDA receptors (GRIN1 and GRIN2B), MAP3K3 and DTNBP1.

## Discussion

Millar et al. [10] originally identified HAPIP, huntingtin-associated protein – interacting protein (also known as Kalirin) as a putative interactor of DISC1, a finding comfirmed by Camargo et al [2] (Network 1), but no direct link to HTT was made. Our updated analysis identifies multiple connections between the DISC1 and HTT pathways and reflects the impact of recent additions to the experimental knowledge base. In addition, three further links involving interactions with intermediate proteins, and not represented in the knowledge-base, have been established between DISC1 and huntingtin. Firstly, the interaction of the schizophrenia candidate gene PCM1 and HAP1, huntingtin-associated protein 1, has been confirmed [11]–[13]. More directly, both N-CoR and GRB2 have been confirmed as interaction partners of both DISC1 and HTT [14]–[18].

The knowledge-based link between DISC1 and HTT highlights possible common modes of action; and suggests that common mechanisms influencing brain vulnerability may be shared between schizophrenia /related psychiatric disorders of complex genetic aetiology and the autosomal dominant disorder of Huntington's disease. Biological support for such a connection comes from the cognitive deficits seen in both disorders, and mouse models. Individuals with mutant huntingtin show deficits in general intelligence tests, memory, and language tasks, even prior to the onset of abnormal motor symptoms [19], [20]. These are key areas of vulnerability in schizophrenia and have been highlighted in studies on psychiatric illness associated with DISC1 [1]. Strikingly, the cognitive deficits seen in a mouse model of Huntington's disease (R6/1) can be rescued by the antipsychotic drug fluoxetine, a potent inhibitor of phosphodiesterase type IV (PDE4A, B, C and D) [21]. Survival rates, hindlimb clasping (a measure of neurological abnormality), and brain atrophy are also significantly reduced by rolipram treatment in the R6/2 mouse model of Huntington's disease [22], [23]. In the Disc1 mutant mouse, L100P, which lies with a missense PDE4B-specific binding site in exon 2, rolipram treatment improves the behavioural and cognitive deficits seen in this model of schizophrenia [24]. Although, rolipram treatment had no effect on the depression-like symptoms seen in the Disc1 Q31L mutant mouse [24]. Interestingly, the major diagnosis in the original t(1∶11) DISC1 translocation family was that of recurrent major depression (10 individuals), as well as schizophrenia (7 individuals) [25]. Patients diagnosed with Huntington's disease show high rates depression, although it is as yet unclear whether this is a co-morbidity or a direct link [26]–[29]. Conversely, individuals with major depression have been shown to have elevated rates of the huntingtin disease-associated alleles (3 allele carriers in 1,000 patients, [30]). However, psychotic schizophrenia-like symptoms have also been noted amongst Huntington's disease patients [31], [32], [33] consistent with the interaction of huntingtin with proteins from other schizophrenia-associated genes such as PCM1, PDE4B and DPYSL2 [11]–[13], [34]–[36]. It is therefore unclear whether the overlap on the effects of disruption of DISC1 and HTT overlap within the domains of depression or schizophrenia.

Indeed, Humbert (2010) has recently suggested that Huntington's disease, like schizophrenia, may have a neurodevelopmental aetiology, with HTT acting as a scaffold protein for many developmental processes critical for correct brain function [36]. Of those that she highlighted, many overlap with known DISC1 functions including: cortical neurogenesis, cell division, regulation of the dynein-dynactin complex, and of β-catenin and the *Wnt* signaling pathway (through regulation of the β-catenin destruction complex). In addition, NMDA receptors appear in both the DISC1 networks in this study suggesting a possible link through NMDA receptor signaling. Huntingtin is a positive transcriptional regulator of other NRSE-containing genes involved in the maintenance of the neuronal phenotype (NRSE, neuron-restrictive silencer element, [37], [38]). Zuccato et al. reported that wild type huntingtin acts on NRSE-containing genes include synaptophysin, NMDA receptor subunits, GABA receptor subunits, acetylcholine receptor alpha 7 and sodium channel genes [37]. Proteins involved in mitochondrial function and/or localisation are identified in the second network as intermediate steps between DISC1 and HTT (i.e. IMMT, DCTN1, DCTN2). Mitochondrial dysfunction in Huntington's disease has recently gained attention [39]–[42]. In Huntington's disease, mutant huntingtin protein induces mitochondrial fragmentation and neuronal cell death [43], [44]. Drp1 mediates mitochondrial fission and is a known GSK3B interacting protein, DISC1 is known to alter GSK3B activity, and overexpression of DISC1 results in a mitochondrial fission/fusion phenotype [45]. It is therefore possible that disruptions of DISC1 or HTT resulting in cognitive deficits are the result of alterations in mitochondrial function. The precise functional overlap between the effects of these genes on liability to mental illness thus awaits experimental validation.

One common limitation of this type of analysis is that the absence of evidence is not evidence of absence. Thus, whereas there is as yet no published evidence that DISC1 and huntingtin interact directly, the number of confirmed interactors that they are seen to have in common makes this a reasonable and testable hypothesis. If demonstrated, this would be give further support for our core hypothesis that the DISC1 and huntingtin pathways are functionally linked. Either way, our updated network analysis provides strong evidence for a link between DISC1 and HTT, and thus implies a degree of biological commonality between the neurological disorders of Huntington's disease and of schizophrenia and related major psychiatric illness. Consideration of this possibility may be instructive in relation to the psychopathological components underlying differential DSM diagnoses and for devising, testing and utilising therapeutic approaches developed primarily for one or the other set of clinical manifestations.

## Methods

The protein list was analysed in Ingenuity Pathway Analysis using the core analysis allowing only direct interactions and with a network size of 140 molecules (run date: 24/05/2010).

Tests for significant enrichment of DISC1 interacting proteins within lists of HTT interacting proteins were performed using the hypergeometric test implemented in Excel.

## Supporting Information

Figure S1 **DISC1 Interactome Network 1.** Only direct interactions were used. Molecules in the input list are in filled in grey.
(JPG)

Figure S2 **DISC1 Interactome Network 2.** Only direct interactions were used. Molecules in the input list are in filled in grey.
(JPG)
