# Supplementary figures and images for: DISC1 and Huntington's Disease – Overlapping Pathways of Vulnerability to Neurological Disorder?
Source: PLoS One. 2011 Jan 26;6(1):e16263. doi: 10.1371/journal.pone.0016263 (PMC3027647; doi:10.1371/journal.pone.0016263)

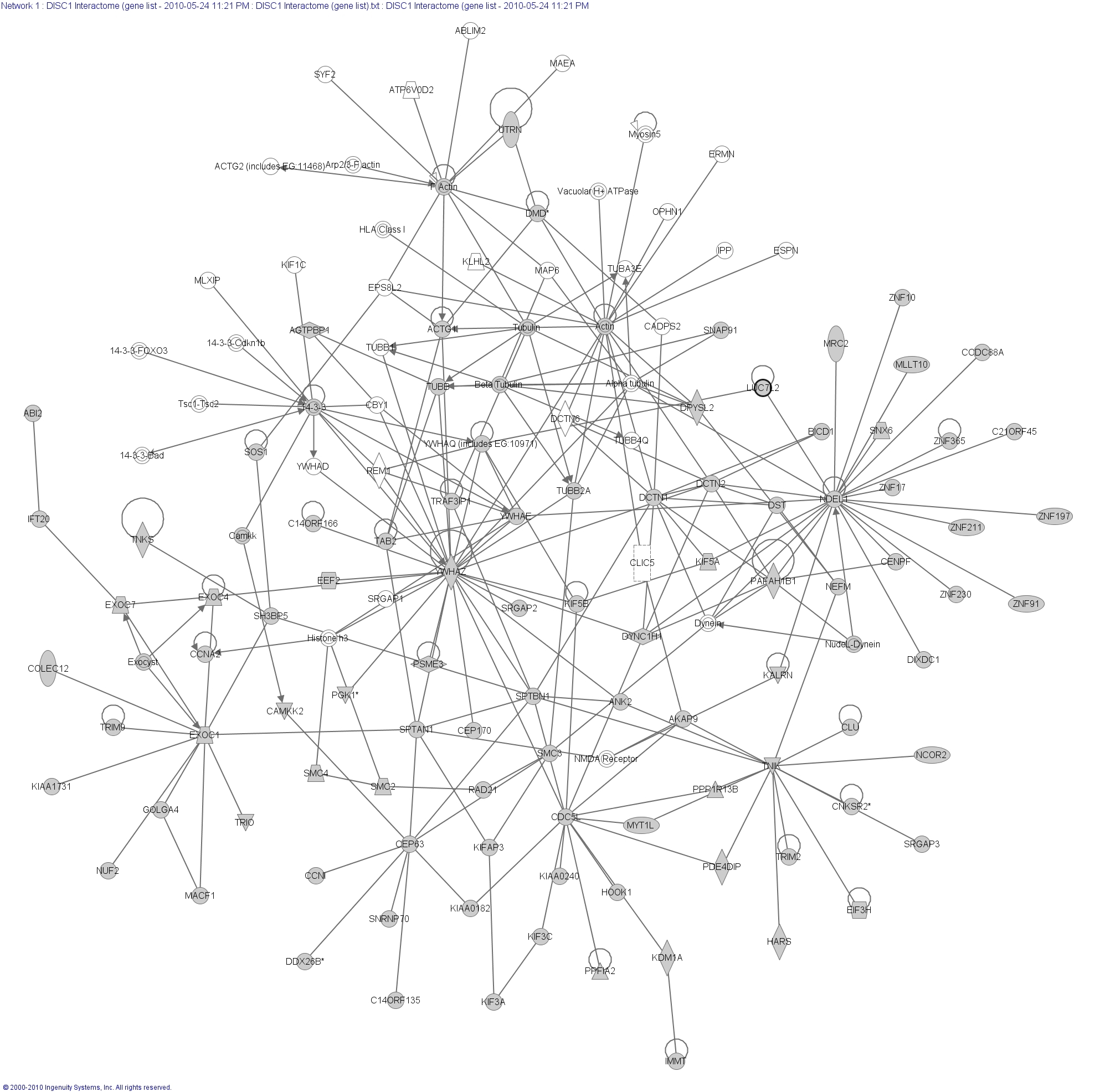

Supplement: Figure S1 — DISC1 Interactome Network 1. Only direct interactions were used. Molecules in the input list are in filled in grey. (JPG) [file pone.0016263.s001.jpg]

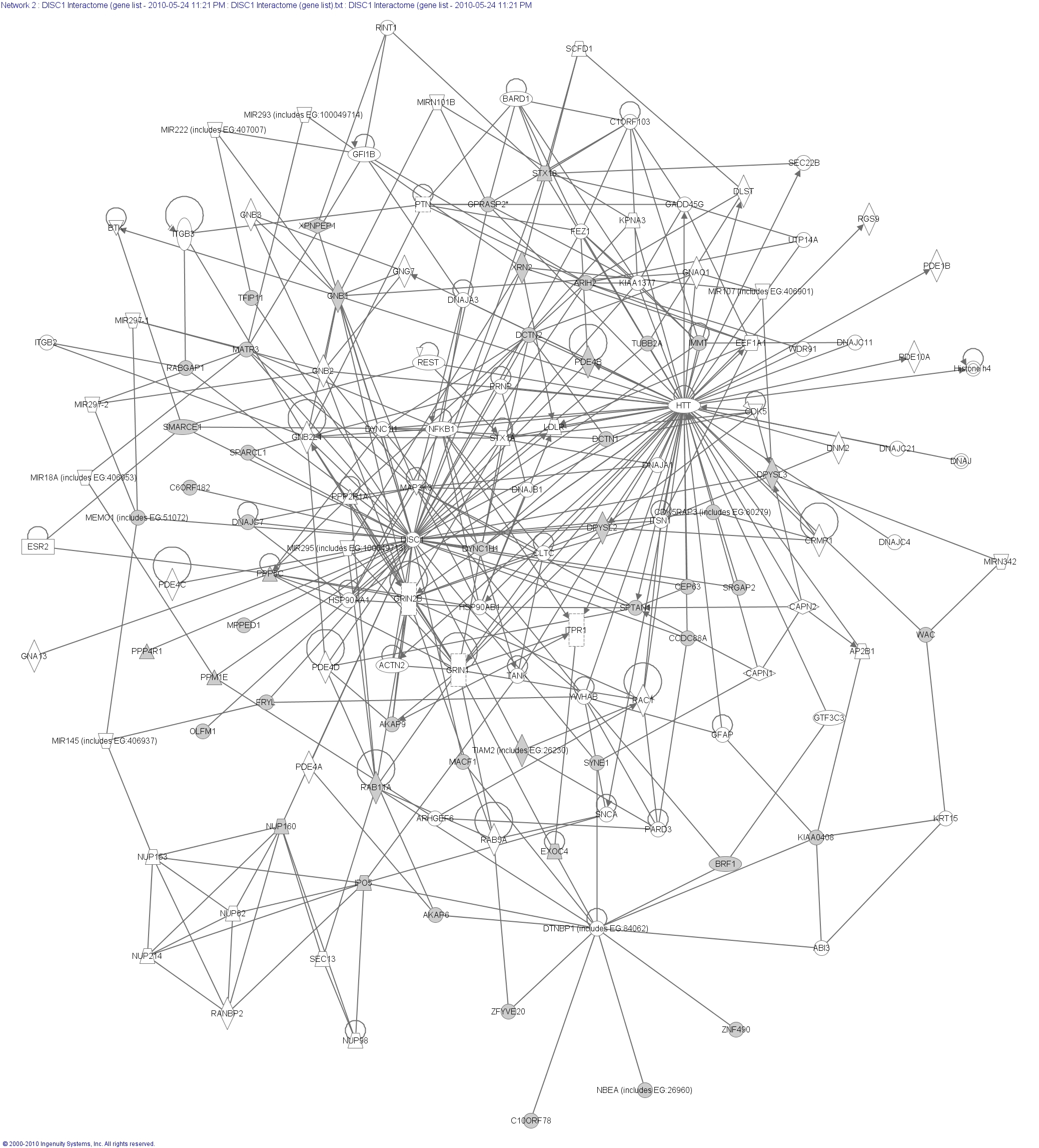

Supplement: Figure S2 — DISC1 Interactome Network 2. Only direct interactions were used. Molecules in the input list are in filled in grey. (JPG) [file pone.0016263.s002.jpg]
